# A global database for conducting systematic reviews and meta-analyses in innovation and quality management

**DOI:** 10.1038/s41597-022-01427-x

**Published:** 2022-06-14

**Authors:** Tibor Csizmadia, Attila Imre Katona

**Affiliations:** 1 University of Pannonia, Department of Management, Veszprém, 8200 Hungary; 2 University of Pannonia, Department of Quantitative Methods, Veszprém, 8200 Hungary

**Keywords:** Databases, Interdisciplinary studies

## Abstract

Innovation and quality management are two fundamental business orientations that complement each other in improving performance and are important drivers of long-term economic growth. These themes have generated widespread attention in the literature; however, most of these studies mainly focused on a narrow area and only in a short term. No systematic effort has been made to build an extended bibliometric database regarding these research areas, which can be immediately used to conduct literature reviews. This study presents a complete (from 1975–2021), up-to-date, preprocessed and geocoded bibliometric database combining published articles of the two themes. The data collection was performed following the PRISMA methodology. The database consists of seven data tables, including one core dataset with 59,231 records and six citation network-related tables, including latitude and longitude values of the affiliations. These data will benefit researchers conducting comparative and in-depth analyses, such as gaining an overview of relevant existing studies, identifying relevant trends and gaining opportunities for a variety of geographic analyses.

## Abstract (summary)

**Table:** 

Measurement(s)	Scientific Publication
Technology Type(s)	Systematic Review
Sample Characteristic - Environment	Innovation and Quality Management
Sample Characteristic - Location	Global

## Background & Summary

In today’s growing global competition, organizations are obliged to promote innovation and improve quality to create, defend and enhance their competitive advantage simultaneously^1–3^. However, a traditional view considers that there is a trade-off between innovation and quality to the extent that the increasing one leads to deteriorating the other^4–6^. Conversely, the modern view rejects this idea and suggests that innovation and quality can coexist together, and companies that achieve excellence in quality are expected to also excel in innovation^7–9^. Thus, innovation and quality management are two necessary business orientations that complement each other in improving performance and are important drivers of long-term economic growth^1,10^.

The significance of innovation research is based on its application across numerous disciplines, the international richness of these studies, and the variety of new ideas, practices, and technologies that have been examined in this field. Moreover, innovation frameworks and theories have become fundamental factors for solving human problems. For example, in a review of COVID-19 vaccine innovations, Vuong *et al*.^11^ demonstrated how COVID-19 vaccines were developed and produced in a very short time. In addition, George *et al*.^12^ explored how digital innovations are helping to tackle climate change and promote sustainable development. Moreover, Toebelmann and Wendler^13^ demonstrated how environmental innovation contributes to reductions in carbon dioxide emissions in EU-27 countries.

Over the past decades, innovation, quality management and their relationship have generated widespread attention in the literature. However, most of these studies mainly focus attention on a narrow area in the short term^14^, and the limited number of studies that have studied the relationship between them shows controversial results^15^. Therefore, to obtain a comprehensive understanding of the context of innovation and quality management, synthesize the extant knowledge on these areas, address relevant gaps and stimulate future research, systematic reviews and meta-analyses need to be performed in the future^16,17^. However, data collection, preprocessing and data mining in systematic reviews and meta-analyses are time- and resource-intensive steps. In addition, most bibliometric platforms provide structured data only without geocoded affiliations, and therefore, it is difficult to analyze research hot spots or hubs. Nevertheless, an open scientific dataset including the aforementioned features and supported by program code can be a powerful tool to encourage open community dialog and support new scientific discoveries^18^.

To our knowledge, no systematic effort has been made to build an extended bibliometric dataset regarding the two research areas, which can be immediately used to conduct literature reviews. Nevertheless, in other research fields, scholars showed that geocoded bibliometric database is strongly valuable^19^. Therefore, we provide an up-to-date and easily accessible cleaned, preprocessed and geocoded bibliometric database combining the published articles of the two themes (innovation and quality management) as a large and comprehensive database.

Our work makes the following significant contributions.

- The database provides a comprehensive overview from 1975 to 2021 of scientific literature in the areas of innovation and quality management.
- Researchers and practitioners can benefit from this database by (1) gaining an overview of relevant existing studies in the fields of innovation and quality management, (2) identifying relevant (future) trends and (3) through geocoded records, gaining opportunities for a variety of geographic distance calculations, spatial clustering and visualizations. In addition, the database allows answering research questions such as “*What kind of relationship can be observed between natural and social sciences in terms of innovation and quality management?*”, “*What spatial-temporal patterns can be observed in the dominant topics related to innovation and/or quality management?*” and “*How the two research fields influenced each other?*
- The data build a baseline for comparative as well as in-depth analyses such as (1) text mining approaches to reveal hidden topics, (2) time-series analyses, (3) citation network analyses, (4) geographical analysis and (5) spatial network analysis.
- The worldwide coverage of the data enables scientific research on innovation and quality management, which supports identifying and analyzing research hot spots and revealing dominant topics from geographical point of view.
- The database can be used to conduct research that supports decision-making in the field of quality management and innovation policy.

The rest of the paper is organized as follows. Section *Methods* describes the methods applied for data collection, preprocessing, data mining and citation network construction in detail, section *Data Records* presents the database structure and provides illustrative examples for using the data associated with text mining approaches, citation network analysis and spatial analysis, section *Technical Validation* shows the validation results and finally, section *Usage Notes* gives data application information.

## Methods

**Fig. 1:**
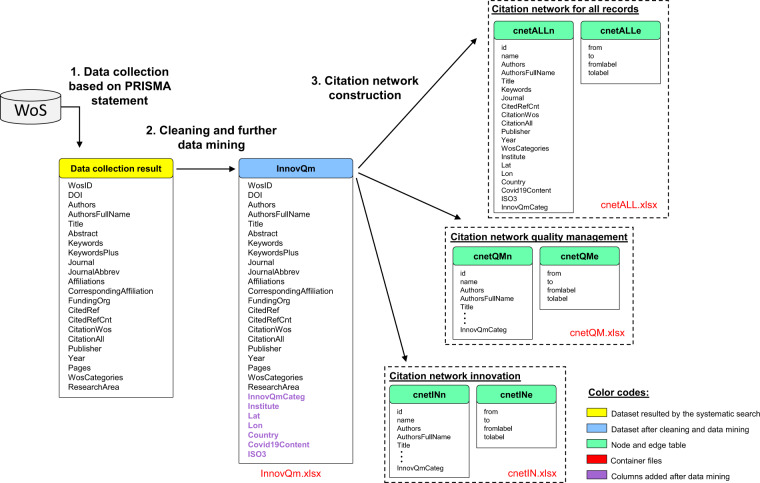
Database construction framework.

Database construction was conducted in three phases: (1) collecting data from the Web of Science (WoS), (2) cleaning and data mining (preprocessing) and (3) constructing citation network nodes and edge tables. Fig. 1 shows the database construction framework.

### Data collection

**Fig. 2:**
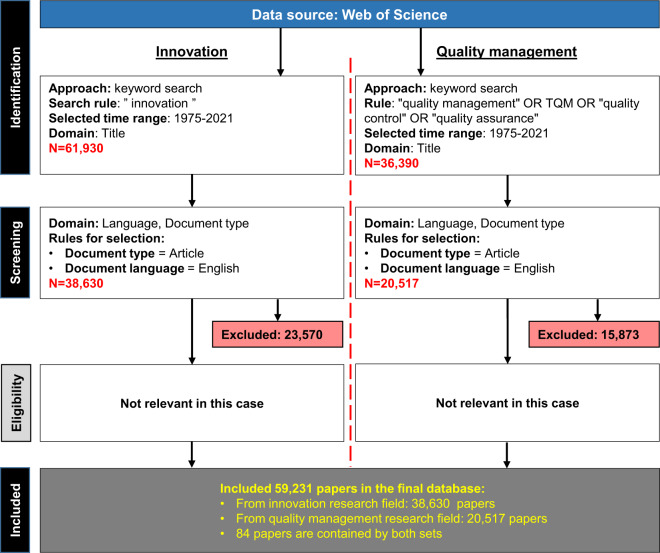
PRISMA flowchart.

Data collection was performed following the PRISMA methodology proposed by^20^. This method provides guidance to scholars in conducting systematic literature reviews by following the four proposed steps: (1) identification, (2) screening, (3) eligibility and (4) included. The PRISMA methodology was selected as the framework of the data collection and filtering due to the following advantages: it provides a comprehensive and transparent process; it is applicable in any research field; and it strongly supports the reproducibility of the review. Furthermore, a process flow diagram (as also contained by PRISMA) helps readers to better understand the overall process and the boundaries of the study and can increase the quality of the literature review^21^. The search was conducted separately for the two areas of interest, and the results were combined to provide the entire bibliometric dataset. Fig. 2 shows the data collection process.

A keyword search was used in both topics on the WoS platform. The search was conducted only on article titles to minimize the inclusion of nonrelevant papers that only mention the terms within the abstract related to innovation or quality management. The entire time range was analyzed (the first available year in WoS was 1975) until the date of data collection, which occurred on 22 September 2021. Regarding the language of the documents, English was considered as the lingua franca of scientific publication^22^. In the case of the innovation field (left side of Fig. 2), 61,930 records were found after the keyword search in the specified time interval. This number was reduced to 38,630 after applying the abovementioned filtering rules by excluding 23,570 papers. In the case of quality management (right side of Fig. 2), 36,390 papers were found by the keyword search, and this number was reduced during the screening phase to 20,517 by excluding 15,873 papers as they were documents with non-English language or were types other than articles. In this paper, step “Eligibility” is not relevant because the number of resulting data records does not make it possible to manually read and evaluate all the screened papers. After the application of the steps proposed by the PRISMA methodology, 59,231 screened papers were collected into the database.

### Cleaning and data mining

The goal of this step was to extend the dataset with additional valuable variables such as the institute of the first author, country of the first author, ISO3 country code, COVID-19 content, geographical coordinates and topic indicator (innovation or quality management). The additional variables were provided based on the following:

#### Institute of the first author (Institute)

The column “*Affiliations*” provided by WoS was used to extract the first author’s affiliation. These data were stored initially as a continuous string including all the authors’ names and affiliations. A further problem was that the same authors from the same affiliation were handled as one entity within the string, and their affiliations did not follow the same format and structure in all cases. Due to this unstructured nature, text cleaning and text mining needed to be used to extract the required information. To return the first author’s affiliation, first, regular expressions were used to remove the unnecessary substrings; second, term shortenings were replaced by their full forms (such as “*University*” instead of “*Univ*.” or “*Department*” instead of “*Dept*.”). Finally, the cleaned string was tokenized to separate the specific parts of the affiliation, such as the institute name, city, street address, and country. These steps were performed using the Python program language.

#### Country-related columns (Country, ISO3)

Using the preprocessed column “*Institute*”, the country of the first author’s affiliation was extracted as part of the cleaned string. Not only were country names extracted, but their ISO3 codes were mapped. Since several statistical programs and packages (such as R) identify countries based on ISO codes, this step makes easy identification possible for statistical packages without the need for further mapping effort by the researcher.

#### COVID-19 content (Covid19Content)

To highlight whether a paper was written in the context of COVID-19, a keyword search was used, and the value of the column was set to 1 if the title, the keywords or the abstract contained at least one of the following keywords: “*COVID*”, “*coronavirus*”, “*pandemic*”, or “*SARSCoV2*”. Otherwise, its value was set to zero.

#### Geographical coordinates (Lat, Lon)

Latitude and longitude values related to the first author’s affiliation were retrieved by geocoding using the *GeoPy* Python package. Geocoding was performed using the extracted and tokenized column “Institution” as input values.

#### Search category indicator (InnovQMCateg)

This column was manually added when combining the results from both searches as described by Fig. 2. If a specific paper was collected exclusively by the innovation-related search, its value was set to “*innovation*”, and the category name “*quality*” indicates that the paper can be found exclusively in the quality management search results. Finally, the intersection was denoted by the category name “*both*”. In this case, duplicated records were removed from the data table.

### Citation network construction

The node and edge tables were generated using the collected and further processed dataset from WoS. In the core dataset, the cited articles were stored in a single column in string format. To construct the edge list format from the string-type input variable, RegEx (regular expressions) commands were applied to find all the DOI numbers appearing within the long text. After extracting the cited DOI numbers, a list format was constructed. The edge list construction process can be described as follows:

1. Select paper *i*
2. Extract all DOI numbers from the string of cited references using regular expressions
3. For all the extracted DOI numbers: add DOI_*i*_ – DOI_*j*_ pairs to the edge list (where *j* is the *j*^*th*^ element of the extracted cited DOIs for paper *i*)
4. Iterate steps 1–3 through all the papers (DOIs) within the core dataset.

The construction was performed in the Python program language using *re*, *NLTK*, *NumPy* and *pandas* packages.

## Data Records

The database (see Excel files with this article) provides an overview from 1975 to 2021 of scientific literature in the areas of innovation and quality management. Table 1 shows the database structure.

**Table 1:** Data tables.

Type	Data table	Description	File	Columns	Rows	Missing (%)
Core dataset	InnovQm	Bibliometric data extracted from WoS extended with geographical information.	InnovQm.xlsx	29	59,321	7.2
Citation network	cnetALLn	Node table of citation network including results of both searches.	cnetALL.xlsx	20	59,178	0.5
Citation network	cnetALLe	Edge table of citation network including results of both searches.	cnetALL.xlsx	4	244,976	<0.01
Citation network	cnetQMn	Node table of citation network including quality management topic.	cnetQM.xlsx	20	20,535	0.4
Citation network	cnetQMe	Edge table of citation network including quality management topic.	cnetQM.xlsx	4	23,511	0
Citation network	cnetINn	Node table of citation network including innovation topic.	cnetIN.xlsx	20	38,645	0.4
Citation network	cnetINe	Edge table of citation network including innovation topic.	cnetIN.xlsx	4	219,925	<0.01

The seven data tables are contained in four Excel files:

- *InnovQm.xlsx*: bibliometric data for each article
- *cnetQM.xlsx*: citation-related network data containing quality management
- *cnetIN.xlsx*: citation-related network data containing innovation
- *cnetALL.xlsx*: citation-related network data containing both fields

All of the four Excel files containing the data tables described in Table 1 are accessible at figshare^23^. The referred figshare database also contains an R notebook file (as well as HTML format) as a related manuscript file (*InnovQmAnalysis.Rmd*) including the visualizations and example analyses such as (1) geographical plotting, (2) co-occurrence network construction, (3) text cleaning and (4) visualization of citation network^23^.

The first file (*InnovQm.xlsx*) shows the bibliometric data for each article, which includes bibliographic information such as authorship, title, year of publication, journal, number of citations and affiliation-related data (see details in Table 2).

**Table 2:** Specification table of InnovQm.xlsx.

Column	Definition	Type	Missing
WosID	ID generated by WoS platform.	char	0.00
DOI	DOI of the articles. In case of missing DOIs, the record number is used.	char	0.00
Authors	Short author names.	char	0.08
AuthorsFullName	Full author names.	char	0.08
Title	Title of the paper.	char	0.00
Abstract	Abstract of the paper.	char	15.75
Keywords	Keywords given by the author(s).	char	33.72
KeywordsPlus	Keywords generated by WoS.	char	34.59
Journal	Full name of the journal.	char	0.00
JournalAbbrev	Abbreviated name of the journal.	char	3.04
Affiliations	Affiliation of each author in string format.	char	12.64
CorrespondingAffiliation	Affiliation of the corresponding author.	char	6.88
FundingOrg	Name of the funding organization.	char	70.64
CitedRef	List of all cited references in string format.	char	6.75
CitedRefCnt	Number of references cited by the paper.	int	0.00
CitationWos	Number of citing references based on WoS core collection.	int	0.00
CitationAll	Number of citing references based on all WoS databases.	int	0.00
Publisher	Publisher of the journal.	char	0.00
Year	Publication year.	int	1.29
Pages	Number of pages as the length of the paper.	int	0.00
WosCategories	Categorization of subject generated by WoS.	char	0.07
ResearchArea	Research area assigned by WoS. Each WoS category is assigned to one research area.	char	0.07
InnovQmCateg	A categorical field that denotes whether the paper was collected by the search related to research field “innovation”, or “quality management”, or contained by both sets.	char	0.00
Institute	Institute of the first author.	char	0.00
Lat	Latitude of the first author’s affiliation.	long	12.64
Lon	Longitude of the first author’s affiliation.	long	12.64
Country	Country of the first author’s affiliation.	char	0.00
Covid19Content	This field denotes if the paper has COVID-19-related content (1 = YES, 0 = NO).	boolean	0.00
ISO3	ISO3 code related to the first author’s country.	char	3.97

**Fig. 3:**
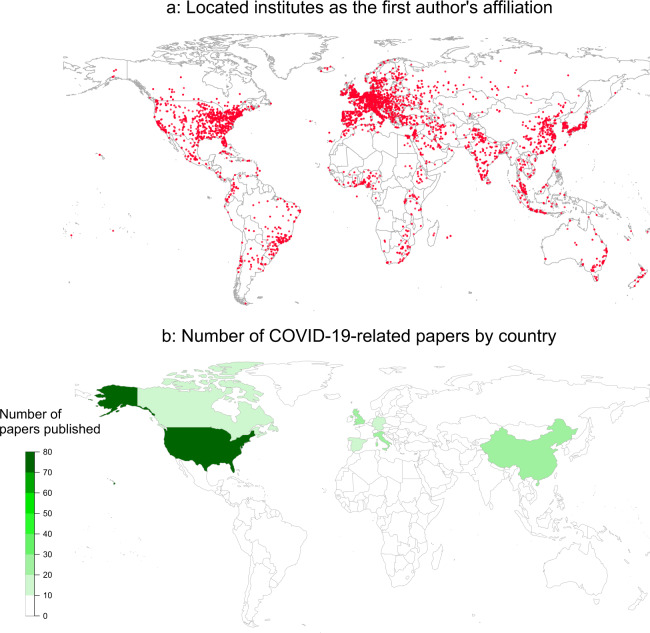
Number of located institutes and COVID-19-related papers in the database.

The InnovQm dataset can be used to conduct several types of analysis, such as text mining on textual fields of bibliometric data (e.g., columns Title, Abstract, Keywords, KeywordsPlus) and geographical analysis using geocoded data (e.g., columns Institute, Country, ISO3). As an example, Fig. 3a shows the 51,743 geocoded institutes as the first author’s affiliation and Fig. 3b depicts the frequency of COVID-19-related papers by country.

**Fig. 4:**
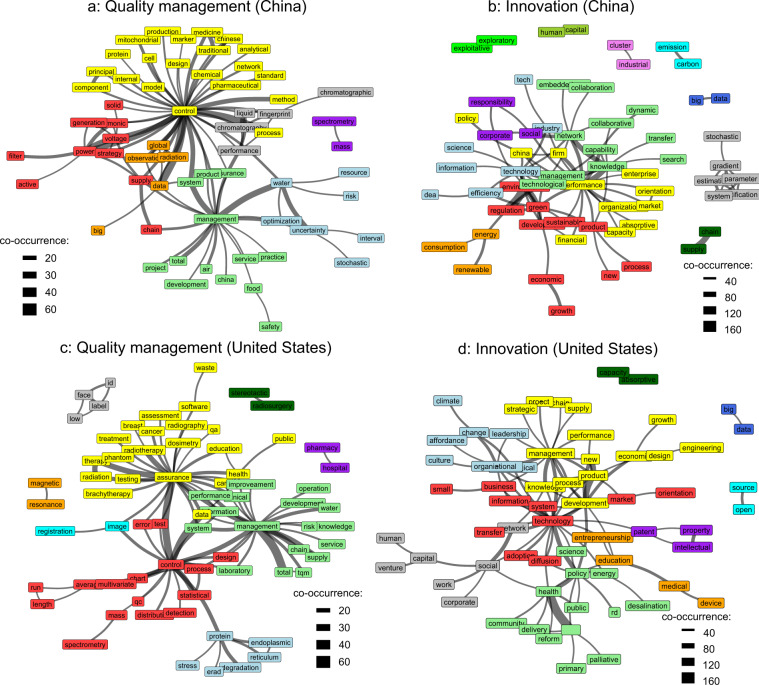
Keyword co-occurrence networks.

As a further example for text analysis, keyword co-occurrences can be visualized by networks, as shown in Fig. 4. The analysis can be conducted by subsetting the dataset based on several factors. Fig. 4 visualizes the co-occurrence network by discipline (using column *InnovQmCateg*) and geographical location (using column *Country*).

The next three Excel files contain citation-related network data connected to quality management (*cnetQM.xlsx*), innovation (*cnetIN.xlsx*) and both fields (*cnetALL.xlsx*). Table 3 shows the structure of the node and edge tables.

**Table 3:** Specification of node and edge tables.

Dataset	Column	Definition	Type	Missing		
				cnetALL	cnetQM	cnetIN
Nodes	id	ID for identification of the nodes in the network. For the identification ID the DOI of the papers are used.	char	0.00	0.00	0.00
	name	A short label generated from the short name of the first author and publication year.	char	0.00	0.00	0.00
	Authors	Short author names.	char	0.14	0.00	0.00
	AuthorsFullName	Full author names.	char	0.14	0.00	0.00
	Title	Title of the paper.	char	0.14	0.00	0.00
	Keywords	Keywords given by the author(s).	char	0.14	0.00	0.00
	Journal	Full name of the journal.	char	0.14	0.00	0.00
	CitedRefCnt	Number of references cited by the paper.	int	0.14	0.00	0.00
	CitationWos	Number of citing references based on the WoS core collection.	int	0.14	0.00	0.00
	CitationAll	Number of citing references based on all WoS databases.	int	0.14	0.00	0.00
	Publisher	Publisher of the journal.	char	0.14	0.00	0.00
	Year	Publication year.	int	0.14	0.00	0.00
	WosCategories	Categorization of subject generated by WoS.	char	0.14	0.00	0.00
	Institute	Institute of the first author.	char	3.80	0.00	0.00
	Lat	Latitude of the first author’s affiliation.	long	12.71	18.51	9.41
	Lon	Longitude of the first author’s affiliation.	long	12.71	18.51	9.41
	Country	Country of the first author’s affiliation.	char	3.80	0.00	0.00
	Covid19Content	This field denotes whether the paper has COVID-19-related content (1 = YES, 0 = NO).	boolean	0.14	0.00	0.00
	ISO3	ISO3 code related to the first author’s country.	char	0.14	0.00	0.00
	InnovQmCateg	A categorical field that denotes whether the paper was collected by the search related to research field “innovation”, or “quality management”, or contained by both sets.	char	0.14	0.00	0.00
Edges	from	ID of the citing node (paper).	char	0.00	0.00	0.00
	to	ID of the cited node (paper).	char	0.00	0.00	0.00
	fromlabel	Short generated label of the citing node (paper).	char	0.00	0.00	0.01
	tolabel	Short generated label of the cited node (paper).	char	0.00	0.00	0.00

**Fig. 5:**
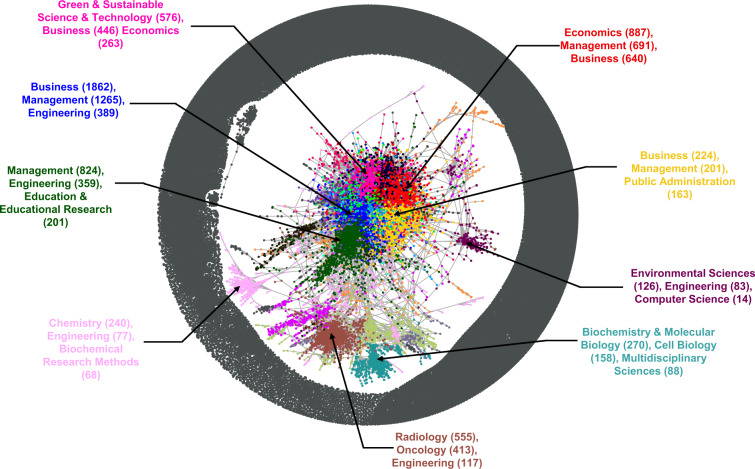
Citation network.

The three node tables are structured according to the scientific papers, which means that one paper is one row. The three edge tables contain the links between the scientific papers indicating citation relationships. It also encloses characteristics such as *Publisher*, *Institute* and *Country*, which can be used for developing different networks. The geocoded data allow us to generate spatial networks. For example, Fig. 5 contains 59,178 nodes and 244,976 edges that are generated by the cnetALL.xlsx file. Using the cnetALL.xlsx file, further networks such as those between authors or institutes can be developed. The variable *Covid19Content* is included in the node tables and provides the opportunity to analyze the subgraph of papers with COVID-19 content.

Fig. 5 shows the citation network of the two themes based on the research fields, where the different colors show different modules as a result of community detection. Labels show the top 3 most frequent WoS categories. As Fig. 5 represents, the different research fields (e.g., green technologies, chemistry, engineering) are well separated, and their relationship within the network can be discovered using the provided database. Further citation networks such as between authors, institutions, countries, research fields, and COVID-19-related topics can be developed. Moreover, each citation network can be constructed in different time intervals since the database covers a long range of publication years.

## Technical Validation

In this section, we present the technical validation of the extracted variables resulting from the additional data mining. First, the geocodes of the affiliations were tested by taking a sample including 287 papers from the dataset. The adequate sample size was determined based on the following equation^24^:

1 $$n=\frac{{Z}_{\alpha /2}^{2}{\sigma }^{2}}{{e}^{2}}$$

where *n* is the sample size, *Z*_*α*/2_ is the Z score of the selected confidence level, *σ* is the standard deviation of the population and *e* is the margin of error during the estimation of the population mean from the sample. The estimation was also performed for latitude and longitude values, and a greater sample size was considered for the sampling strategy:

2 $$n=max\left\{{n}_{Longitude},{n}_{Latitude}\right\}=max\left\{\frac{{Z}_{\alpha /2}^{2}{\sigma }_{Latitude}^{2}}{{e}_{Latitude}^{2}},\frac{{Z}_{\alpha /2}^{2}{\sigma }_{Longitude}^{2}}{{e}_{Longitude}^{2}}\right\}$$

**Fig. 6:**
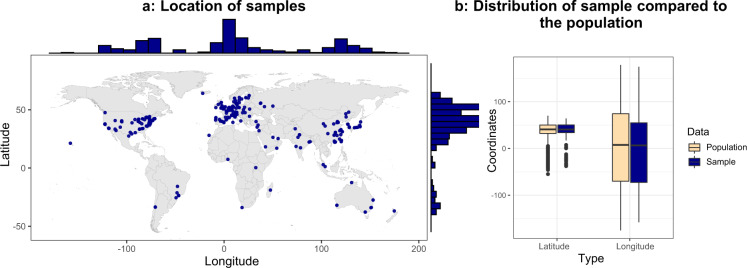
Sample properties.

During the calculation, the confidence level was set to 95%, which indicates 1.96 as the Z score value. To represent an overall 5% error interval, *e* was set to ±2.5% of the given latitude/longitude ranges. After the substitution of the adequate values, the sample size can be determined as *n* = *max*{185.80, 286.79}, indicating that 287 samples should be taken. The sample was randomly taken from the dataset, and Fig. 6 illustrates the geographical locations and distribution of the sample compared to the population.

As the histograms in Fig. 6a show, the sample covers a good range of geographical locations, including all continents. It is also observable that the sample well describes the mean and range of the population (see Fig. 6b).

**Fig. 7:**
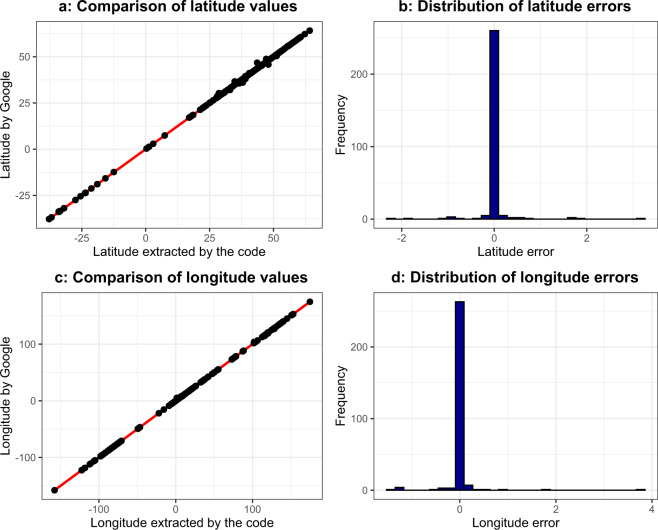
Validation of the geographical coordinates.

Fig. 7a,c show the relationship between the automatically extracted (by the Python code) and the manually searched coordinates. The red reference line includes those points where the results of the manual search and data extraction were equal. The subfigures show a good fitting and strong relationship with a 0.99 (Pearson) correlation coefficient in both cases. Fig. 7b,d show the distribution of the errors (the difference between the expected value and the extracted value). The mean absolute deviation (MAD) is 0.08 in the case of latitudes, and its value is 0.07 in the case of longitudes. Two cases were found with higher differences in terms of longitudes, but after manual checking, we concluded that they did not cause significant differences in the geographical pattern. Based on the above, it can be concluded that the performance of geocoding is acceptable.

The institution names, country names, country codes and validity of the cited references were checked in relation to the selected 287 papers. During the examination, manual checking was performed, and no mistakes were found. The existence of citations was validated using Google Scholar.

## Usage Notes

Our database can be used to conduct literature reviews and meta-analyses in the field of innovation and/or quality management. It is also possible to analyze the relationship between the two research fields. Text mining methods can be applied, such as topic mining, word clouds, and co-occurrence networks, to reveal the trends in latent topics. Using citation networks, the most important hubs can be identified with the application of a wide range of network centrality metrics. It is also possible to construct multilayer networks (such as applied by Gadár *et al*.^25^) to analyze the relationship between the two fields, and adding publication time as a dimension enables us to model the evolution of these research fields. Due to the geocoded variables, spatial analysis can also be conducted, even with spatial citation networks, to identify geographical patterns.

### Limitations

It is necessary to note that the proposed database has some limitations.

- The last step of the PRISMA methodology (“Eligibility”) could not be performed due to the data size; therefore, PRISMA methodology was partly followed.
- Global bibliographic databases can have some shortcomings. In the Web of Science dataset used, some DOI numbers are missing, which can cause some citations to go undetected.
- As Vuong *et al*.^26^ pointed out, bibliographic datasets can include different name versions for the same author. Although this problem does not affect the examples provided in this paper, the users should take this into consideration when applying author-level analyses.
- Finally, despite being indexed in global bibliometric databases, in some cases, published papers can be retracted^27^, and this may not be reflected in the developed datasets.

## Data Availability

Data preprocessing tasks were performed in Python programming language. A Jupyter notebook (Geocode_cnet.ipynb) including the data preprocessing steps is provided alongside the paper^23^. Example figures (geographical distribution, co-occurrence networks, citation network) were constructed in the R program language. The R script is provided to produce and modify the figures based on the needs of the researcher.
